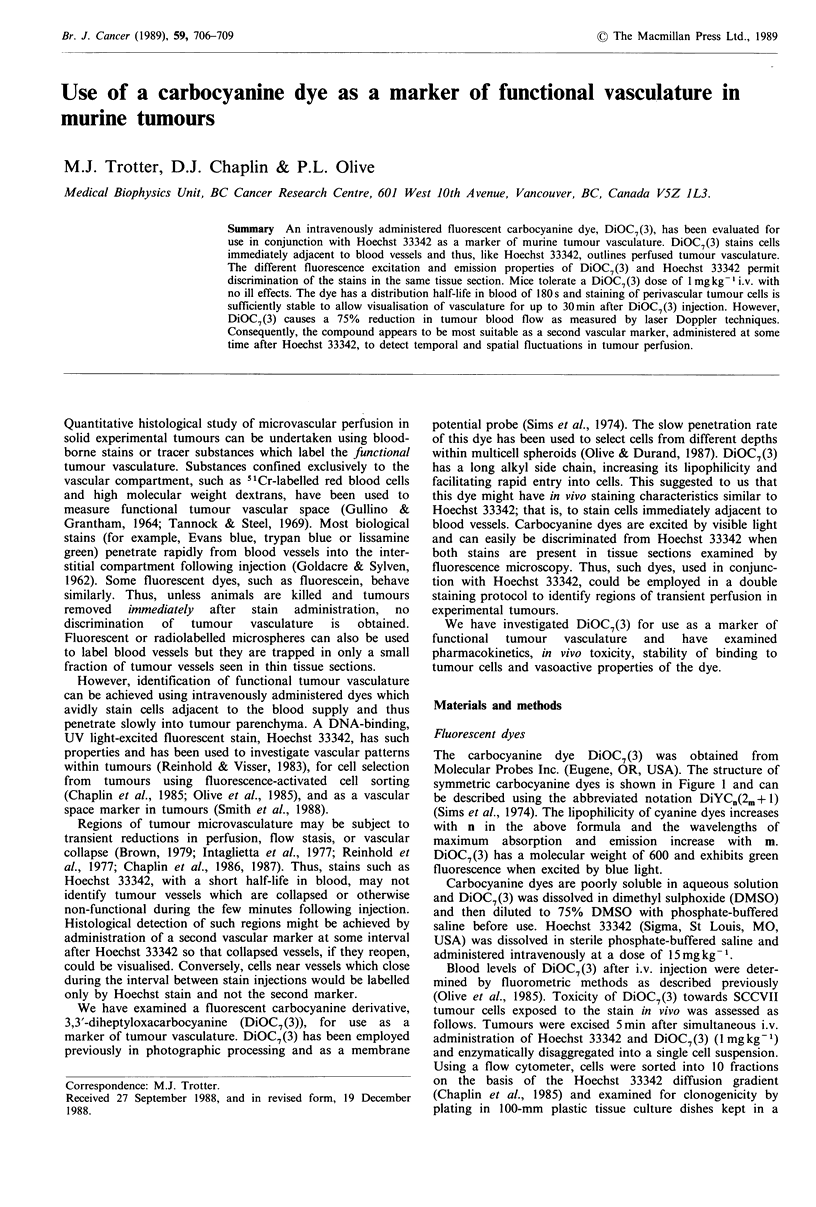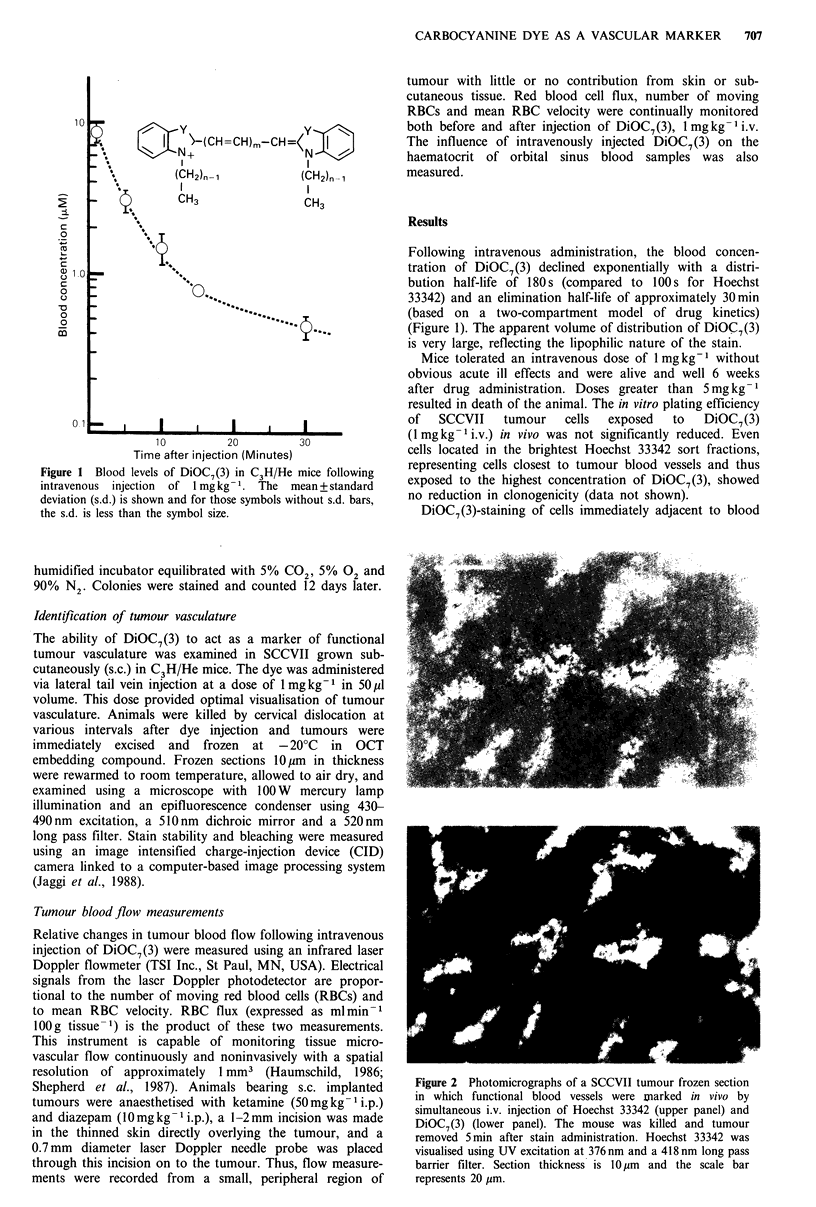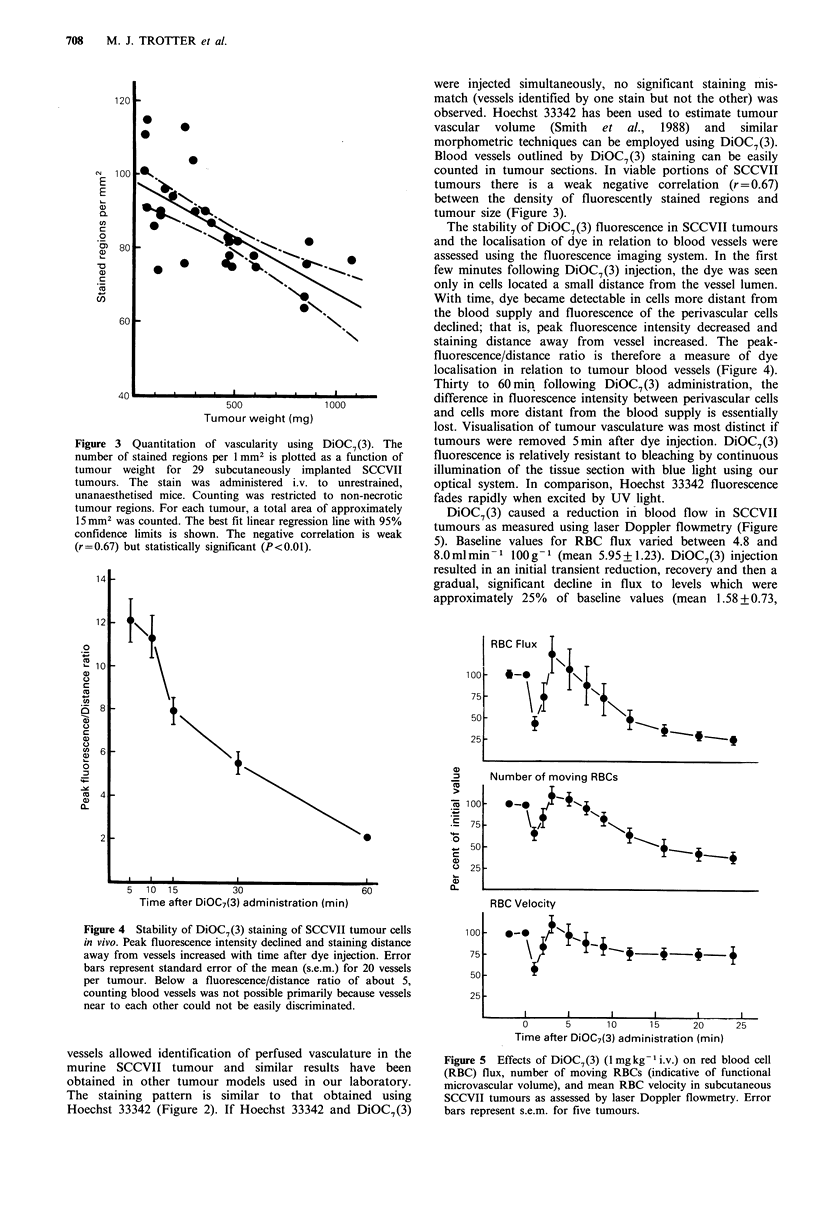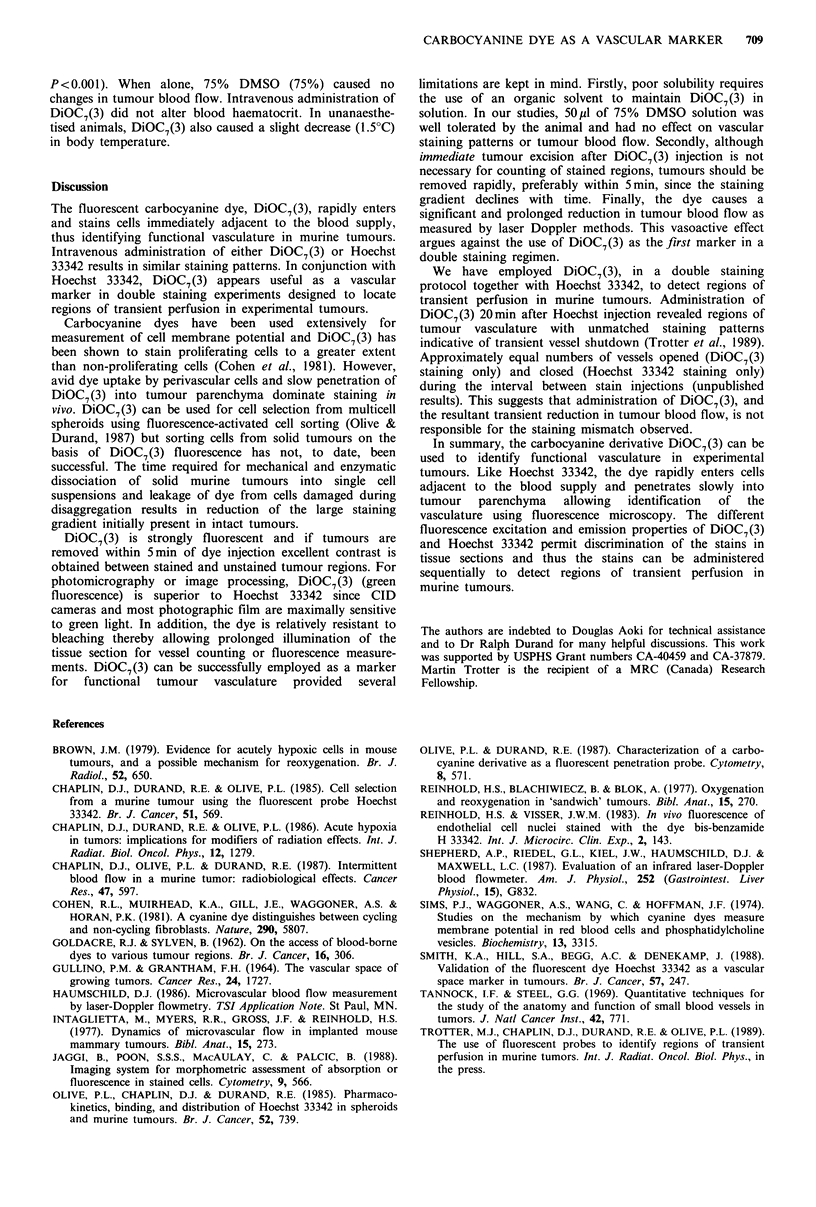# Use of a carbocyanine dye as a marker of functional vasculature in murine tumours.

**DOI:** 10.1038/bjc.1989.148

**Published:** 1989-05

**Authors:** M. J. Trotter, D. J. Chaplin, P. L. Olive

**Affiliations:** Medical Biophysics Unit, BC Cancer Research Centre, Vancouver, Canada.

## Abstract

**Images:**


Br. J. Cancer (1989), 59, 706–709 © The Macmillan Press Ltd., 1989

### Use of a carbocyanine dye as a marker of functional vasculature in murine tumours

M.J. Trotter, D.J. Chaplin & P.L. Olive

*Medical Biophysics Unit, BC Cancer Research Centre, 601 West 10th Avenue, Vancouver, BC, Canada V5Z 1L3.*

**Summary** An intravenously administered fluorescent carbocyanine dye, $DiOC_7(3)$, has been evaluated for use in conjunction with Hoechst 33342 as a marker of murine tumour vasculature. $DiOC_7(3)$ stains cells immediately adjacent to blood vessels and thus, like Hoechst 33342, outlines perfused tumour vasculature. The different fluorescence excitation and emission properties of $DiOC_7(3)$ and Hoechst 33342 permit discrimination of the stains in the same tissue section. Mice tolerate a $DiOC_7(3)$ dose of $1\,mg\,kg^{-1}$ i.v. with no ill effects. The dye has a distribution half-life in blood of 180 s and staining of perivascular tumour cells is sufficiently stable to allow visualisation of vasculature for up to 30 min after $DiOC_7(3)$ injection. However, $DiOC_7(3)$ causes a 75% reduction in tumour blood flow as measured by laser Doppler techniques. Consequently, the compound appears to be most suitable as a second vascular marker, administered at some time after Hoechst 33342, to detect temporal and spatial fluctuations in tumour perfusion.

Quantitative histological study of microvascular perfusion in solid experimental tumours can be undertaken using blood-borne stains or tracer substances which label the *functional* tumour vasculature. Substances confined exclusively to the vascular compartment, such as $^{51}Cr$-labelled red blood cells and high molecular weight dextrans, have been used to measure functional tumour vascular space (Gullino & Grantham, 1964; Tannock & Steel, 1969). Most biological stains (for example, Evans blue, trypan blue or lissamine green) penetrate rapidly from blood vessels into the interstitial compartment following injection (Goldacre & Sylven, 1962). Some fluorescent dyes, such as fluorescein, behave similarly. Thus, unless animals are killed and tumours removed *immediately* after stain administration, no discrimination of tumour vasculature is obtained. Fluorescent or radiolabelled microspheres can also be used to label blood vessels but they are trapped in only a small fraction of tumour vessels seen in thin tissue sections.

However, identification of functional tumour vasculature can be achieved using intravenously administered dyes which avidly stain cells adjacent to the blood supply and thus penetrate slowly into tumour parenchyma. A DNA-binding, UV light-excited fluorescent stain, Hoechst 33342, has such properties and has been used to investigate vascular patterns within tumours (Reinhold & Visser, 1983), for cell selection from tumours using fluorescence-activated cell sorting (Chaplin *et al.*, 1985; Olive *et al.*, 1985), and as a vascular space marker in tumours (Smith *et al.*, 1988).

Regions of tumour microvasculature may be subject to transient reductions in perfusion, flow stasis, or vascular collapse (Brown, 1979; Intaglietta *et al.*, 1977; Reinhold *et al.*, 1977; Chaplin *et al.*, 1986, 1987). Thus, stains such as Hoechst 33342, with a short half-life in blood, may not identify tumour vessels which are collapsed or otherwise non-functional during the few minutes following injection. Histological detection of such regions might be achieved by administration of a second vascular marker at some interval after Hoechst 33342 so that collapsed vessels, if they reopen, could be visualised. Conversely, cells near vessels which close during the interval between stain injections would be labelled only by Hoechst stain and not the second marker.

We have examined a fluorescent carbocyanine derivative, 3,3′-diheptyloxacarbocyanine ($DiOC_7(3)$), for use as a marker of tumour vasculature. $DiOC_7(3)$ has been employed previously in photographic processing and as a membrane potential probe (Sims *et al.*, 1974). The slow penetration rate of this dye has been used to select cells from different depths within multicell spheroids (Olive & Durand, 1987). $DiOC_7(3)$ has a long alkyl side chain, increasing its lipophilicity and facilitating rapid entry into cells. This suggested to us that this dye might have *in vivo* staining characteristics similar to Hoechst 33342; that is, to stain cells immediately adjacent to blood vessels. Carbocyanine dyes are excited by visible light and can easily be discriminated from Hoechst 33342 when both stains are present in tissue sections examined by fluorescence microscopy. Thus, such dyes, used in conjunction with Hoechst 33342, could be employed in a double staining protocol to identify regions of transient perfusion in experimental tumours.

We have investigated $DiOC_7(3)$ for use as a marker of functional tumour vasculature and have examined pharmacokinetics, *in vivo* toxicity, stability of binding to tumour cells and vasoactive properties of the dye.

### Materials and methods

### *Fluorescent dyes*

The carbocyanine dye $DiOC_7(3)$ was obtained from Molecular Probes Inc. (Eugene, OR, USA). The structure of symmetric carbocyanine dyes is shown in Figure 1 and can be described using the abbreviated notation $DiYC_n(2_m+1)$ (Sims *et al.*, 1974). The lipophilicity of cyanine dyes increases with **n** in the above formula and the wavelengths of maximum absorption and emission increase with **m**. $DiOC_7(3)$ has a molecular weight of 600 and exhibits green fluorescence when excited by blue light.

Carbocyanine dyes are poorly soluble in aqueous solution and $DiOC_7(3)$ was dissolved in dimethyl sulphoxide (DMSO) and then diluted to 75% DMSO with phosphate-buffered saline before use. Hoechst 33342 (Sigma, St Louis, MO, USA) was dissolved in sterile phosphate-buffered saline and administered intravenously at a dose of $15\,mg\,kg^{-1}$.

Blood levels of $DiOC_7(3)$ after i.v. injection were determined by fluorometric methods as described previously (Olive *et al.*, 1985). Toxicity of $DiOC_7(3)$ towards SCCVII tumour cells exposed to the stain *in vivo* was assessed as follows. Tumours were excised 5 min after simultaneous i.v. administration of Hoechst 33342 and $DiOC_7(3)$ ($1\,mg\,kg^{-1}$) and enzymatically disaggregated into a single cell suspension. Using a flow cytometer, cells were sorted into 10 fractions on the basis of the Hoechst 33342 diffusion gradient (Chaplin *et al.*, 1985) and examined for clonogenicity by plating in 100-mm plastic tissue culture dishes kept in a

Correspondence: M.J. Trotter.
Received 27 September 1988, and in revised form, 19 December 1988.

**Figure 1** Blood levels of $DiOC_7(3)$ in $C_3H/He$ mice following intravenous injection of $1 mg kg^{-1}$. The mean ± standard deviation (s.d.) is shown and for those symbols without s.d. bars, the s.d. is less than the symbol size.

humidified incubator equilibrated with 5% $CO_2$, 5% $O_2$ and 90% $N_2$. Colonies were stained and counted 12 days later.

*Identification of tumour vasculature*

The ability of $DiOC_7(3)$ to act as a marker of functional tumour vasculature was examined in SCCVII grown subcutaneously (s.c.) in $C_3H/He$ mice. The dye was administered via lateral tail vein injection at a dose of $1 mg kg^{-1}$ in 50 μl volume. This dose provided optimal visualisation of tumour vasculature. Animals were killed by cervical dislocation at various intervals after dye injection and tumours were immediately excised and frozen at −20°C in OCT embedding compound. Frozen sections 10 μm in thickness were rewarmed to room temperature, allowed to air dry, and examined using a microscope with 100 W mercury lamp illumination and an epifluorescence condenser using 430–490 nm excitation, a 510 nm dichroic mirror and a 520 nm long pass filter. Stain stability and bleaching were measured using an image intensified charge-injection device (CID) camera linked to a computer-based image processing system (Jaggi *et al.*, 1988).

*Tumour blood flow measurements*

Relative changes in tumour blood flow following intravenous injection of $DiOC_7(3)$ were measured using an infrared laser Doppler flowmeter (TSI Inc., St Paul, MN, USA). Electrical signals from the laser Doppler photodetector are proportional to the number of moving red blood cells (RBCs) and to mean RBC velocity. RBC flux (expressed as $ml min^{-1} 100 g tissue^{-1}$) is the product of these two measurements. This instrument is capable of monitoring tissue microvascular flow continuously and noninvasively with a spatial resolution of approximately $1 mm^3$ (Haumschild, 1986; Shepherd *et al.*, 1987). Animals bearing s.c. implanted tumours were anaesthetised with ketamine ($50 mg kg^{-1}$ i.p.) and diazepam ($10 mg kg^{-1}$ i.p.), a 1–2 mm incision was made in the thinned skin directly overlying the tumour, and a 0.7 mm diameter laser Doppler needle probe was placed through this incision on to the tumour. Thus, flow measurements were recorded from a small, peripheral region of tumour with little or no contribution from skin or subcutaneous tissue. Red blood cell flux, number of moving RBCs and mean RBC velocity were continually monitored both before and after injection of $DiOC_7(3)$, $1 mg kg^{-1}$ i.v. The influence of intravenously injected $DiOC_7(3)$ on the haematocrit of orbital sinus blood samples was also measured.

### Results

Following intravenous administration, the blood concentration of $DiOC_7(3)$ declined exponentially with a distribution half-life of 180 s (compared to 100 s for Hoechst 33342) and an elimination half-life of approximately 30 min (based on a two-compartment model of drug kinetics) (Figure 1). The apparent volume of distribution of $DiOC_7(3)$ is very large, reflecting the lipophilic nature of the stain.

Mice tolerated an intravenous dose of $1 mg kg^{-1}$ without obvious acute ill effects and were alive and well 6 weeks after drug administration. Doses greater than $5 mg kg^{-1}$ resulted in death of the animal. The *in vitro* plating efficiency of SCCVII tumour cells exposed to $DiOC_7(3)$ ($1 mg kg^{-1}$ i.v.) *in vivo* was not significantly reduced. Even cells located in the brightest Hoechst 33342 sort fractions, representing cells closest to tumour blood vessels and thus exposed to the highest concentration of $DiOC_7(3)$, showed no reduction in clonogenicity (data not shown).

$DiOC_7(3)$-staining of cells immediately adjacent to blood

**Figure 2** Photomicrographs of a SCCVII tumour frozen section in which functional blood vessels were marked *in vivo* by simultaneous i.v. injection of Hoechst 33342 (upper panel) and $DiOC_7(3)$ (lower panel). The mouse was killed and tumour removed 5 min after stain administration. Hoechst 33342 was visualised using UV excitation at 376 nm and a 418 nm long pass barrier filter. Section thickness is 10 μm and the scale bar represents 20 μm.

**Figure 3** Quantitation of vascularity using $DiOC_7(3)$. The number of stained regions per $1\,mm^2$ is plotted as a function of tumour weight for 29 subcutaneously implanted SCCVII tumours. The stain was administered i.v. to unrestrained, unanaesthetised mice. Counting was restricted to non-necrotic tumour regions. For each tumour, a total area of approximately $15\,mm^2$ was counted. The best fit linear regression line with 95% confidence limits is shown. The negative correlation is weak ($r=0.67$) but statistically significant ($P<0.01$).

**Figure 4** Stability of $DiOC_7(3)$ staining of SCCVII tumour cells *in vivo*. Peak fluorescence intensity declined and staining distance away from vessels increased with time after dye injection. Error bars represent standard error of the mean (s.e.m.) for 20 vessels per tumour. Below a fluorescence/distance ratio of about 5, counting blood vessels was not possible primarily because vessels near to each other could not be easily discriminated.

vessels allowed identification of perfused vasculature in the murine SCCVII tumour and similar results have been obtained in other tumour models used in our laboratory. The staining pattern is similar to that obtained using Hoechst 33342 (Figure 2). If Hoechst 33342 and $DiOC_7(3)$ were injected simultaneously, no significant staining mismatch (vessels identified by one stain but not the other) was observed. Hoechst 33342 has been used to estimate tumour vascular volume (Smith *et al.*, 1988) and similar morphometric techniques can be employed using $DiOC_7(3)$. Blood vessels outlined by $DiOC_7(3)$ staining can be easily counted in tumour sections. In viable portions of SCCVII tumours there is a weak negative correlation ($r=0.67$) between the density of fluorescently stained regions and tumour size (Figure 3).

The stability of $DiOC_7(3)$ fluorescence in SCCVII tumours and the localisation of dye in relation to blood vessels were assessed using the fluorescence imaging system. In the first few minutes following $DiOC_7(3)$ injection, the dye was seen only in cells located a small distance from the vessel lumen. With time, dye became detectable in cells more distant from the blood supply and fluorescence of the perivascular cells declined; that is, peak fluorescence intensity decreased and staining distance away from vessel increased. The peak-fluorescence/distance ratio is therefore a measure of dye localisation in relation to tumour blood vessels (Figure 4). Thirty to 60 min following $DiOC_7(3)$ administration, the difference in fluorescence intensity between perivascular cells and cells more distant from the blood supply is essentially lost. Visualisation of tumour vasculature was most distinct if tumours were removed 5 min after dye injection. $DiOC_7(3)$ fluorescence is relatively resistant to bleaching by continuous illumination of the tissue section with blue light using our optical system. In comparison, Hoechst 33342 fluorescence fades rapidly when excited by UV light.

$DiOC_7(3)$ caused a reduction in blood flow in SCCVII tumours as measured using laser Doppler flowmetry (Figure 5). Baseline values for RBC flux varied between 4.8 and $8.0\,ml\,min^{-1}\,100\,g^{-1}$ (mean $5.95 \pm 1.23$). $DiOC_7(3)$ injection resulted in an initial transient reduction, recovery and then a gradual, significant decline in flux to levels which were approximately 25% of baseline values (mean $1.58 \pm 0.73$,

**Figure 5** Effects of $DiOC_7(3)$ ($1\,mg\,kg^{-1}$ i.v.) on red blood cell (RBC) flux, number of moving RBCs (indicative of functional microvascular volume), and mean RBC velocity in subcutaneous SCCVII tumours as assessed by laser Doppler flowmetry. Error bars represent s.e.m. for five tumours.

$P<0.001$). When alone, 75% DMSO (75%) caused no changes in tumour blood flow. Intravenous administration of $DiOC_7(3)$ did not alter blood haematocrit. In unanaesthetised animals, $DiOC_7(3)$ also caused a slight decrease (1.5°C) in body temperature.

### Discussion

The fluorescent carbocyanine dye, $DiOC_7(3)$, rapidly enters and stains cells immediately adjacent to the blood supply, thus identifying functional vasculature in murine tumours. Intravenous administration of either $DiOC_7(3)$ or Hoechst 33342 results in similar staining patterns. In conjunction with Hoechst 33342, $DiOC_7(3)$ appears useful as a vascular marker in double staining experiments designed to locate regions of transient perfusion in experimental tumours.

Carbocyanine dyes have been used extensively for measurement of cell membrane potential and $DiOC_7(3)$ has been shown to stain proliferating cells to a greater extent than non-proliferating cells (Cohen *et al.*, 1981). However, avid dye uptake by perivascular cells and slow penetration of $DiOC_7(3)$ into tumour parenchyma dominate staining *in vivo*. $DiOC_7(3)$ can be used for cell selection from multicell spheroids using fluorescence-activated cell sorting (Olive & Durand, 1987) but sorting cells from solid tumours on the basis of $DiOC_7(3)$ fluorescence has not, to date, been successful. The time required for mechanical and enzymatic dissociation of solid murine tumours into single cell suspensions and leakage of dye from cells damaged during disaggregation results in reduction of the large staining gradient initially present in intact tumours.

$DiOC_7(3)$ is strongly fluorescent and if tumours are removed within 5 min of dye injection excellent contrast is obtained between stained and unstained tumour regions. For photomicrography or image processing, $DiOC_7(3)$ (green fluorescence) is superior to Hoechst 33342 since CID cameras and most photographic film are maximally sensitive to green light. In addition, the dye is relatively resistant to bleaching thereby allowing prolonged illumination of the tissue section for vessel counting or fluorescence measurements. $DiOC_7(3)$ can be successfully employed as a marker for functional tumour vasculature provided several limitations are kept in mind. Firstly, poor solubility requires the use of an organic solvent to maintain $DiOC_7(3)$ in solution. In our studies, 50 $\mu$l of 75% DMSO solution was well tolerated by the animal and had no effect on vascular staining patterns or tumour blood flow. Secondly, although *immediate* tumour excision after $DiOC_7(3)$ injection is not necessary for counting of stained regions, tumours should be removed rapidly, preferably within 5 min, since the staining gradient declines with time. Finally, the dye causes a significant and prolonged reduction in tumour blood flow as measured by laser Doppler methods. This vasoactive effect argues against the use of $DiOC_7(3)$ as the *first* marker in a double staining regimen.

We have employed $DiOC_7(3)$, in a double staining protocol together with Hoechst 33342, to detect regions of transient perfusion in murine tumours. Administration of $DiOC_7(3)$ 20 min after Hoechst injection revealed regions of tumour vasculature with unmatched staining patterns indicative of transient vessel shutdown (Trotter *et al.*, 1989). Approximately equal numbers of vessels opened ($DiOC_7(3)$ staining only) and closed (Hoechst 33342 staining only) during the interval between stain injections (unpublished results). This suggests that administration of $DiOC_7(3)$, and the resultant transient reduction in tumour blood flow, is not responsible for the staining mismatch observed.

In summary, the carbocyanine derivative $DiOC_7(3)$ can be used to identify functional vasculature in experimental tumours. Like Hoechst 33342, the dye rapidly enters cells adjacent to the blood supply and penetrates slowly into tumour parenchyma allowing identification of the vasculature using fluorescence microscopy. The different fluorescence excitation and emission properties of $DiOC_7(3)$ and Hoechst 33342 permit discrimination of the stains in tissue sections and thus the stains can be administered sequentially to detect regions of transient perfusion in murine tumours.

The authors are indebted to Douglas Aoki for technical assistance and to Dr Ralph Durand for many helpful discussions. This work was supported by USPHS Grant numbers CA-40459 and CA-37879. Martin Trotter is the recipient of a MRC (Canada) Research Fellowship.

### References

BROWN, J.M. (1979). Evidence for acutely hypoxic cells in mouse tumours, and a possible mechanism for reoxygenation. *Br. J. Radiol.*, **52**, 650.

CHAPLIN, D.J., DURAND, R.E. & OLIVE, P.L. (1985). Cell selection from a murine tumour using the fluorescent probe Hoechst 33342. *Br. J. Cancer*, **51**, 569.

CHAPLIN, D.J., DURAND, R.E. & OLIVE, P.L. (1986). Acute hypoxia in tumors: implications for modifiers of radiation effects. *Int. J. Radiat. Biol. Oncol. Phys.*, **12**, 1279.

CHAPLIN, D.J., OLIVE, P.L. & DURAND, R.E. (1987). Intermittent blood flow in a murine tumor: radiobiological effects. *Cancer Res.*, **47**, 597.

COHEN, R.L., MUIRHEAD, K.A., GILL, J.E., WAGGONER, A.S. & HORAN, P.K. (1981). A cyanine dye distinguishes between cycling and non-cycling fibroblasts. *Nature*, **290**, 5807.

GOLDACRE, R.J. & SYLVEN, B. (1962). On the access of blood-borne dyes to various tumour regions. *Br. J. Cancer*, **16**, 306.

GULLINO, P.M. & GRANTHAM, F.H. (1964). The vascular space of growing tumors. *Cancer Res.*, **24**, 1727.

HAUMSCHILD, D.J. (1986). Microvascular blood flow measurement by laser-Doppler flowmetry. *TSI Application Note*. St Paul, MN.

INTAGLIETTA, M., MYERS, R.R., GROSS, J.F. & REINHOLD, H.S. (1977). Dynamics of microvascular flow in implanted mouse mammary tumours. *Bibl. Anat.*, **15**, 273.

JAGGI, B., POON, S.S.S., MACAULAY, C. & PALCIC, B. (1988). Imaging system for morphometric assessment of absorption or fluorescence in stained cells. *Cytometry*, **9**, 566.

OLIVE, P.L., CHAPLIN, D.J. & DURAND, R.E. (1985). Pharmacokinetics, binding, and distribution of Hoechst 33342 in spheroids and murine tumours. *Br. J. Cancer*, **52**, 739.

OLIVE, P.L. & DURAND, R.E. (1987). Characterization of a carbocyanine derivative as a fluorescent penetration probe. *Cytometry*, **8**, 571.

REINHOLD, H.S., BLACHIWIECZ, B. & BLOK, A. (1977). Oxygenation and reoxygenation in 'sandwich' tumours. *Bibl. Anat.*, **15**, 270.

REINHOLD, H.S. & VISSER, J.W.M. (1983). *In vivo* fluorescence of endothelial cell nuclei stained with the dye bis-benzamide H 33342. *Int. J. Microcirc. Clin. Exp.*, **2**, 143.

SHEPHERD, A.P., RIEDEL, G.L., KIEL, J.W., HAUMSCHILD, D.J. & MAXWELL, L.C. (1987). Evaluation of an infrared laser-Doppler blood flowmeter. *Am. J. Physiol.*, **252** (*Gastrointest. Liver Physiol.*, **15**), G832.

SIMS, P.J., WAGGONER, A.S., WANG, C. & HOFFMAN, J.F. (1974). Studies on the mechanism by which cyanine dyes measure membrane potential in red blood cells and phosphatidylcholine vesicles. *Biochemistry*, **13**, 3315.

SMITH, K.A., HILL, S.A., BEGG, A.C. & DENEKAMP, J. (1988). Validation of the fluorescent dye Hoechst 33342 as a vascular space marker in tumours. *Br. J. Cancer*, **57**, 247.

TANNOCK, I.F. & STEEL, G.G. (1969). Quantitative techniques for the study of the anatomy and function of small blood vessels in tumors. *J. Natl Cancer Inst.*, **42**, 771.

TROTTER, M.J., CHAPLIN, D.J., DURAND, R.E. & OLIVE, P.L. (1989). The use of fluorescent probes to identify regions of transient perfusion in murine tumors. *Int. J. Radiat. Oncol. Biol. Phys.*, in the press.